# Comparative Analysis of Next-Generation Sequencing and Immunohistochemistry in MSI/MMR Testing*

**DOI:** 10.5146/tjpath.2025.14079

**Published:** 2025-09-30

**Authors:** Cisel Aydın Mericoz, Zeynep Secil Satılmıs, Fatma Esrefı, Gulsum Caylak, Burcu Saka, Ayse Armutlu, Orhun Cig Taskın, Ibrahim Kulac

**Affiliations:** Department of Pathology, School of Medicine, Koç University, Istanbul, Türkiye; Koç University Hospital, Istanbul, Türkiye; Department of Pathology, School of Medicine, Koç University, Istanbul, Türkiye; KUIS Artificial Intelligence Center, Istanbul, Türkiye; Research Center for Translational Medicine, Koç University, Istanbul, Türkiye

**Keywords:** Next-generation sequencing, Microsatellite instability, Microsatellite stable, Mismatch repair

## Abstract

***Objective: *** Loss of mismatch repair (MMR) protein expression, assessed via immunohistochemistry (IHC), and microsatellite instability (MSI) status, determined through molecular methods, are two tumor-agnostic predictive biomarkers for immunotherapy eligibility. However, there remains no consensus on the preferred testing method, nor on the type and extent of molecular testing required for optimal patient selection. This study investigates the correlation between MMR protein loss detected by IHC and MSI status identified through next-generation sequencing (NGS) to evaluate the concordance and potential complementary roles of these methods.

***Material and Methods:*** A total of 139 tumor samples were analyzed for MSI using NGS. The cohort included colorectal carcinoma (n=51), pancreatic ductal adenocarcinoma (n=22), cholangiocarcinoma (n=9), non-small cell lung carcinoma (n=6), adenoid cystic carcinoma (n=6), gastric adenocarcinoma (n=6), high-grade serous ovarian carcinoma (n=5), and 34 other tumor types. IHC was performed to assess MLH1, MSH2, MSH6, and PMS2 protein expression. The correlation between MSI status and MMR protein loss was evaluated.

***Results: *** Twelve tumors (8.6%) were classified as MSI-High (microsatellite instable). Among them, ten exhibited MMR protein loss, whereas two MSI-High tumors (a mucinous adenocarcinoma of omental origin and a mucinous colon adenocarcinoma) retained MMR protein expression. No MMR-deficient tumors were identified as MSI-Low (microsatellite stable/MSS).

***Conclusion:*** A strong correlation exists between IHC-based MMR loss and NGS-based MSI detection. IHC remains widely used due to its accessibility and cost-effectiveness, whereas NGS offers higher accuracy and broader genomic insights. With its ability to detect multiple alterations simultaneously, NGS is particularly valuable when tissue is scarce. Combining both methods can improve diagnostic accuracy and guide optimal immunotherapy selection.

## INTRODUCTION

The DNA mismatch repair (MMR) pathway is a critical mechanism for maintaining genomic stability by correcting base substitutions and insertion-deletion (indel) errors during DNA replication (1). Loss of function in one or more MMR proteins, through genetic or epigenetic mechanisms, results in MMR deficiency (dMMR), leading to increased mutation rates and contributing to tumorigenesis (2). Microsatellite instability (MSI), a hallmark of dMMR, is characterized by alterations in short tandem repeat sequences and serves as a tumor-agnostic predictive biomarker for response to immune checkpoint inhibitors, such as pembrolizumab (3).

MSI is recognized as one of the key agnostic biomarkers for predicting response to immunotherapy (4). The evaluation of MMR protein loss through IHC has been widely utilized by pathologists as a reliable, in-situ diagnostic method (5). Immunohistochemistry (IHC), which evaluates the expression of MLH1, MSH2, MSH6, and PMS2 proteins, remains the most widely used method for detecting MMR deficiency, and therefore MSI status indirectly. While IHC is cost-effective and accessible, indeterminate cases cause confusion and some cases with no MMR loss may be MSI-High due to other mechanisms that IHC would miss (6–8). An alternative to IHC is the molecular detection of MSI. Traditional polymerase chain reaction (PCR)-based methods analyze size variations in a small panel of microsatellite markers (9). However, these methods face challenges in cancers outside of colorectal and endometrial origins, for which they were primarily designed (10,11). Additionally, PCR-based assays are limited by the small number of loci analyzed, tissue- and population-specific variability and the absence of matched normal samples can impact their sensitivity and specificity (9,12). More recently, next-generation sequencing (NGS)-based approaches have gained global attention, offering high sensitivity and the ability to provide detailed molecular insights (3,13–15). NGS-based MSI detection offers a more comprehensive approach by analyzing a larger number of microsatellite regions in addition to other genomic variants (14,16). NGS has demonstrated the potential to improve MSI detection accuracy across various tumor types, even in the absence of matched normal samples, if the right cut-off values are determined (17). However, challenges remain, including inter- and intra-tumoral heterogeneity and population-level polymorphisms in microsatellite regions, which can affect analytical performance.

Beyond MSI detection, NGS provides additional genomic insights by identifying single nucleotide variants (SNVs), copy number variations (CNVs), and other molecular alterations that may have diagnostic, prognostic, or therapeutic implications. Genomic characterization of colorectal, endometrial or pancreatic tumors has become an essential approach for oncological management. The comprehensive profiling capability makes NGS particularly valuable in settings where tissue availability is limited, such as small biopsy specimens and cytological samples (18–20). In these cases, where traditional IHC and PCR-based MSI assessments may be challenging due to insufficient material, NGS allows for a broader molecular characterization while simultaneously evaluating MSI status.

Although MSI/MMR testing is widely used in clinical practice, there is still no clear consensus on the optimal testing method—whether IHC or NGS—especially across different tumor types. Most studies to date have focused on colorectal and endometrial cancers, with fewer data available for other malignancies.

In this study, we assessed the concordance between IHC-based MMR protein expression and NGS-based MSI status in a cohort predominantly composed of colorectal and pancreatic ductal adenocarcinomas, along with selected other tumor types. We aimed to assess the level of agreement between these methods in routine diagnostic settings and to highlight rare discordant cases where NGS may offer additional insight.

## MATERIALS and METHODS

### Study Design and Sample Selection

This retrospective study included all tumor samples analyzed at our institution between January 2023 and June 2024 for which both MMR IHC and NGS-based MSI results were available. The cohort comprised a diverse set of cancer types, including 51 colorectal carcinomas (49 adenocarcinomas, 1 medullary carcinoma, 1 small cell carcinoma), 22 pancreatic ductal adenocarcinomas, 9 cholangiocarcinomas, 6 non-small cell lung carcinomas, 6 adenoid cystic carcinomas, 6 gastric adenocarcinomas, 5 high-grade serous ovarian carcinomas, and 34 other tumor types. The sample size was determined by the total number of eligible cases within this time frame; no formal power calculation was conducted due to the descriptive nature of the study.

**Inclusion criteria** were: (i) histologically confirmed malignant tumors; (ii) availability of interpretable IHC results for MLH1, MSH2, MSH6, and PMS2; and (iii) successful MSI assessment by one of the three NGS platforms described.

**Exclusion criteria** included: (i) incomplete or indeterminate IHC staining; (ii) inadequate tissue quality for NGS; or (iii) missing data regarding MSI classification.

### Immunohistochemistry

To evaluate MMR protein expression, 4-µm thick sections from formalin-fixed, paraffin-embedded (FFPE) tumor samples were used for IHC. Antibodies targeting MLH1 (ES05, Dako, Mouse monoclonal), MSH2 (FE11, Dako, Mouse monoclonal), MSH6 (EP49, Dako, Rabbit monoclonal), PMS2 (EP51, Dako, Rabbit monoclonal) were applied using the Dako OMNIS automated staining system. The internal positive control included non-tumoral cells within the same tissue section. Tumor cells were considered to have retained expression if nuclear staining was observed and was comparable to that of internal non-tumoral controls. Complete absence of nuclear staining in tumor cells, in the presence of intact staining in adjacent normal cells, was interpreted as loss of expression. This cohort did not have any case with indeterminate MMR staining on IHC.

### Next-Generation Sequencing (NGS)

NGS-based MSI testing was conducted using three different assays: VariantPlex® Solid Tumor Focus v2, AVENIO® Comprehensive Genomic Profiling (CGP) Kit, and Illumina TruSight® Oncology 500 (TSO-500). Of the 139 tumor samples, 57 cases were analyzed by VariantPlex® Solid Tumor Focus v2, 65 cases by the AVENIO® CGP Kit, and 17 cases by TSO-500.

The VariantPlex® Solid Tumor Focus v2 panel (ArcherDx) analyzes 20 cancer-related genes and approximately 108–111 microsatellite loci. It determines MSI status based on the fraction of unstable loci. A sample is classified as MSI-High (microsatellite instable) if more than 30% of loci are unstable, and as MSS (microsatellite stable/MSI-Low) if less than 20% are unstable. If 20–30% of loci are unstable, the sample is considered MSI-Intermediate and further evaluation with an orthogonal method is recommended.

The AVENIO® Tumor Tissue Comprehensive Genomic Profiling (CGP) Kit (Roche) targets 324 genes and reports three genomic signatures: MSI, TMB, and genomic loss of heterozygosity (gLOH). MSI is determined through a proprietary algorithm, with a predefined threshold of ≥0.0124 used to classify cases as MSI-High (AVENIO Tumor Tissue CGP Kit V2 Data Sheet). The number of assessed microsatellite loci is not disclosed.

The TruSight® Oncology 500 (TSO-500) assay (Illumina) covers 523 genes and evaluates MSI and TMB using a hybrid-capture strategy. Approximately 130 microsatellite loci are assessed, with at least 40 evaluable loci required for MSI calling (TruSight Oncology 500 Data Sheet). MSI status is reported as the proportion of unstable loci and benchmarked against a reference dataset.

DNA was extracted from FFPE tissue blocks, and sequencing libraries were prepared following validated protocols. Bioinformatics pipelines analyzed the microsatellite regions for instability, with MSI status classified as high (MSI-H / microsatellite-instable) or low (MSI-L / microsatellite-stable / MSS) based on predefined thresholds. For consistency, the terms ‘microsatellite instable’ and ‘MSI-High/MSI-H’ are used interchangeably throughout the manuscript. Similarly, ‘microsatellite stable’, ‘MSS’, and ‘MSI-Low’ are used synonymously.

### Statistical Analysis

Concordance between MSI status and MMR protein expression was evaluated using both percentage agreement and Cohen’s Kappa coefficient to assess inter-method reliability. In addition, Fisher’s exact test was performed to examine the association between MSI classification (MSI-High vs. MSS) and MMR status (dMMR vs. MMRp). A p-value less than 0.05 was considered statistically significant. All analyses were descriptive and exploratory in nature.

## RESULTS

### Overall Concordance Between IHC and NGS

**Figure 1:**
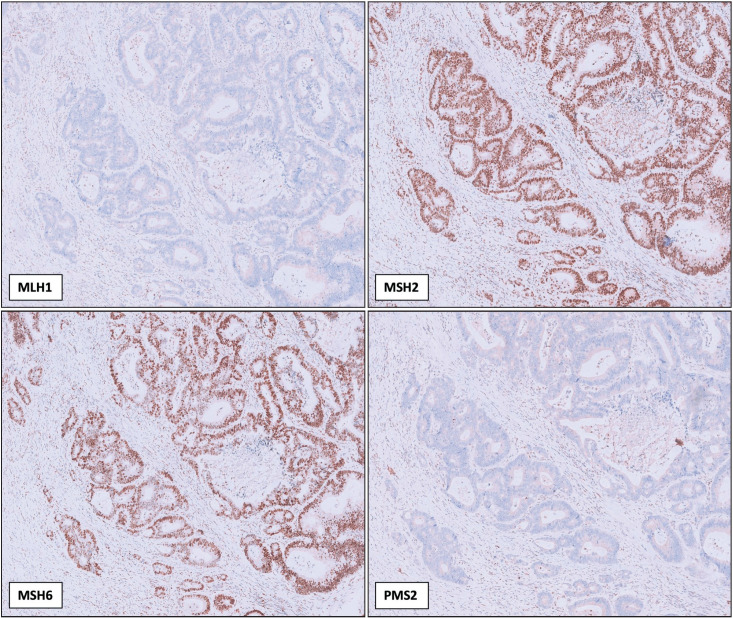
Immunohistochemical staining results of a tumor exhibiting MLH1 and PMS2 loss, with intact MSH2 and MSH6 expression

Out of the 139 tumor samples analyzed, 12 (10%) cases were detected MSI-H by NGS. Of these, 10 cases exhibited loss of one or more MMR protein expression on IHC, resulting in a concordance rate of 91% for MSI-H tumors (Table 1). Among the MSI-H and dMMR cases, 7 showed concurrent MLH1 and PMS2 protein loss (Figure 1), 1 case exhibited only MLH1 loss, 1 case demonstrated MSH2 and MSH6 loss, and 1 case showed isolated MSH6 loss (Table 2) (Among the remaining 127 MSS tumors, all retained MMR protein expression, indicating a 100% concordance in MSS cases. The overall concordance rate between IHC and NGS for all tumor samples was 99.0%.

**Table 1:** Cross table of MSI Status (NGS) and MMR Status (IHC)

		**Immunohistochemistry**		
		**dMMR**	**MMRp**	**Total**
Next Generation Sequencing	MSI-High	10	2	12
	MSS	0	127	127
	Total	10	129	139

**Table 2:** MMR Protein Expression and *BRAF* Mutation Status in MSI-High Cases

**Diagnosis**	**Age**	**MS status**	**MMR status**	**MLH1 loss**	**MSH2 loss**	**MSH6 loss**	**PMS2 loss**	***BRAF*** **status**
Endometrial carcinoma	65	MSI -High	dMMR	Deficient	Retained	Retained	Retained	*BRAF* wild
Endometrial carcinoma	71	MSI -High	dMMR	Deficient	Retained	Retained	Deficient	*BRAF* wild
Colon adenocarcinoma	46	MSI -High	dMMR	Deficient	Retained	Retained	Deficient	*BRAF* wild
Colon adenocarcinoma	80	MSI -High	dMMR	Deficient	Retained	Retained	Deficient	*BRAF* V600E mutant
Colon adenocarcinoma	83	MSI -High	dMMR	Deficient	Retained	Retained	Deficient	*BRAF* V600E mutant
Colon adenocarcinoma	57	MSI -High	dMMR	Deficient	Retained	Retained	Deficient	*BRAF* wild
Endometrial carcinoma	59	MSI -High	dMMR	Deficient	Retained	Retained	Deficient	*BRAF* wild
Colon adenocarcinoma	51	MSI -High	dMMR	Retained	Deficient	Deficient	Retained	*BRAF* wild
Colon medullary carcinoma	76	MSI -High	dMMR	Deficient	Retained	Retained	Deficient	*BRAF* wild
Colon adenocarcinoma	45	MSI -High	MMRp	Retained	Retained	Retained	Retained	*BRAF* wild
Colon adenocarcinoma	48	MSI -High	dMMR	Retained	Retained	Deficient	Retained	*BRAF* wild
Adenocarcinoma, NOS	47	MSI -High	MMRp	Retained	Retained	Retained	Retained	*BRAF* wild

Statistical analysis demonstrated a near-perfect agreement between NGS-based MSI detection and IHC-based MMR evaluation, with a Cohen’s Kappa coefficient of 0.93. However, this result should be interpreted carefully since our data has an asymmetrical distribution. A Fisher’s exact test comparing MSI status and MMR expression revealed a statistically significant association (p < 0.0001).

### Details of MSI-High Tumors

**Figure 2:**
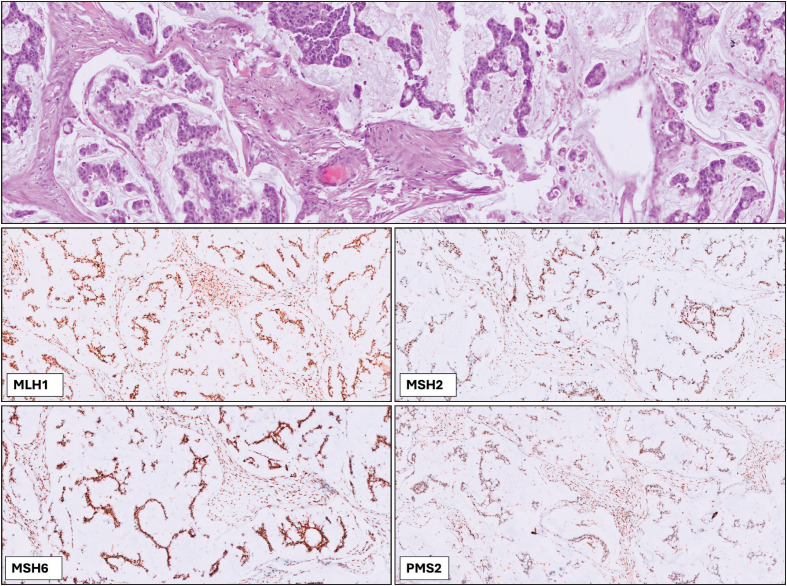
Representative H&E and immunohistochemistry images of an MSI-High case without MMR protein loss. The H&E-stained section shows the histopathological features of the tumor, while IHC staining for MLH1, MSH2, MSH6, and PMS2 demonstrates intact protein expression.

Among the 12 MSI-High cases identified, 8 were colorectal carcinomas (including 1 medullary carcinoma of the colon and 1 mucinous adenocarcinoma), 3 were endometrial carcinomas, and 1 was a tumor of unknown primary origin (Table 2). Of these, 2 cases (2/12, 16%) were MSI-MMR discordant: one was a mucinous adenocarcinoma of colorectal origin, with 56 out of 109 regions (51.38%) identified as unstable, and the other was a mucinous adenocarcinoma of unknown origin, with 84 out of 108 regions (73.68%) identified as unstable (Figure 2). These cases were analyzed using the VariantPlex® Solid Tumor Focus v2 panel.

Among these 12 MSI-High tumors, 2 cases harbored the *BRAF* p.V600E mutation. Notably, both of these *BRAF*-mutant cases also exhibited dMMR.

### Panel-Specific Findings

For samples analyzed using the VariantPlex® Solid Tumor Focus v2 panel, two discordant results were observed, where the tumor was identified MSI-High by NGS, but no MMR protein loss was identified on IHC. No discordant cases were identified with the AVENIO® CGP Kit and TSO-500 assays. In all MSS cases evaluated by the ArcherDx VariantPlex® Solid Tumor Focus v2 panel, AVENIO® CGP Kit, and TSO-500, MMR protein expression was confirmed as retained by IHC. This consistency underscores the reliability of these NGS panels in identifying MSS tumors and correlating them with intact MMR protein status.

## DISCUSSION

This study evaluated the concordance between IHC-based MMR protein expression and NGS-based MSI status across a cohort of 139 tumor samples. We found an overall agreement rate of 99.0%, with only two discordant cases out of twelve MSI-High tumors. These results support the clinical reliability of both testing methods in routine diagnostics.

Statistical analysis showed a near-perfect agreement between the two methods, with a Cohen’s Kappa coefficient of 0.93 and a highly significant association by Fisher’s exact test (p < 0.0001). Our findings are consistent with the literature, which generally reports strong concordance between IHC and NGS-based MSI assessment. Bartels et al. (21) demonstrated high agreement across tumor types, and Ali-Fehmi et al. (22) confirmed the robustness of both methods in a series of over 19,000 tumors. Additionally, Kang et al. (23) reported a Kappa coefficient of 0.91 in a large endometrial carcinoma cohort, further supporting the reliability of concordance between NGS and IHC in clinical settings. Of the twelve MSI-High cases in our cohort, two showed intact MMR protein expression by IHC, representing a discordance rate of 16.7% among MSI-H tumors. While rare, such discordant cases have been described in previous studies and may result from various mechanisms. One possibility is the presence of missense mutations that lead to the expression of nonfunctional MMR proteins (24), which can retain antigenicity and therefore stain positive on IHC. However, the interpretation of MMR immunostaining requires careful attention to subtle patterns such as focal, weak, or dot-like staining, which may be misclassified as “retained” expression and lead to under recognition of dMMR cases (5,15,25). Additionally, alterations in genes outside the canonical MMR pathway—such as *POLE* or *POLD1*—have been reported to result in hypermutation and microsatellite instability without detectable loss of MMR protein expression (26–28). Moreover, mutations in noncanonical MMR-related genes, including *MSH3*, *PMS1*, and *EPCAM*, can contribute to MSI-H phenotypes without corresponding loss of the four primary MMR proteins detectable by IHC (29). For instance, *EPCAM* deletions can lead to *MSH2* promoter hypermethylation, resulting in MMR deficiency without direct *MSH2* gene mutations (30). In our cohort, both discordant cases were mucinous adenocarcinomas, and neither harbored a *POLE* mutation, making *MLH1* promoter methylation less likely. Although we were unable to perform further molecular characterization due to sample limitations and financial constraints, these cases underline the potential value of NGS in detecting MSI that may be missed by IHC alone.

Among the MSI-High cases in our cohort, two tumors harbored a *BRAF* p.V600E mutation. Both of these were associated with concurrent MLH1 and PMS2 protein loss, supporting the interpretation of a sporadic origin via *MLH1* promoter hypermethylation (31,32). In contrast, the two MSI-H tumors that retained MMR protein expression were *BRAF* wild-type, which further reduces the likelihood of *MLH1* methylation and supports the hypothesis of alternative mechanisms underlying MSI in these cases. This distinction underscores the importance of integrating *BRAF* mutational status into the evaluation of MSI-H tumors, particularly for differentiating sporadic from potentially hereditary cases (33). In addition to MSI detection, NGS offers the advantage of simultaneous assessment of clinically relevant genomic alterations, including *BRAF, KRAS*, and *POLE* mutations, as well as tumor mutational burden (TMB). This is particularly important in small biopsy specimens, where tissue availability is limited. In our study, comprehensive genomic profiling was achieved using only a single 20-μm section, in contrast to the multiple sections typically required for separate PCR and IHC analyses.

A potential limitation of our study is the use of three different NGS panels, each with its own gene content, number of microsatellite loci, and MSI classification thresholds (17,21,34,35). Commercial platforms such as the AVENIO® Tumor Tissue CGP Kit and the TruSight® Oncology 500 (TSO-500) come with their own pre-validated MSI algorithms and thresholds, whereas in-house assays often require custom validation. For instance, some studies have proposed cut-off values such as MSI-H (≥20%), borderline MSI (≥7% and <20%), and MSS (<7%) based on instability scores across loci (17).

Notably, both of the discordant cases in our cohort—MSI-H by NGS but with intact MMR expression by IHC—were identified using the VariantPlex® panel. Although this may suggest increased sensitivity of this assay in certain contexts, the sample size is insufficient to draw firm conclusions. Still, it raises an important point regarding inter-platform variability in MSI detection. While many studies have compared IHC and NGS methods, few have directly compared NGS panels among themselves. Recent work by Adams et al. (35) underscores the value of such comparisons, highlighting differences in locus number, capture methods, and thresholds across platforms. Future standardization efforts should consider not only cross-platform agreement with IHC or PCR, but also internal consistency among NGS-based MSI detection tools.

The high concordance observed in our study reinforces the reliability of both IHC and NGS-based MSI testing in clinical practice. While IHC remains a cost-effective and widely accessible method, NGS provides additional molecular insights, making it especially valuable in contexts where tissue is limited, or comprehensive genomic profiling is required. The identification of rare discordant cases further highlights the complementary role of NGS, particularly in tumors with atypical morphology or equivocal staining.

From a therapeutic perspective, accurate MSI detection is critical, as MSI-High status is an established predictive biomarker for response to immune checkpoint inhibitors such as pembrolizumab (4). Ensuring the correct identification of MSI-H tumors—particularly those that may be missed by one method (36)—has direct implications for immunotherapy eligibility and patient outcomes. Future research should aim to standardize MSI detection thresholds across platforms, validate findings in larger and tumor-specific cohorts, and explore the functional impact of noncanonical mechanisms leading to MSI. Comparative studies focused on NGS panel performance in real-world settings may also help optimize assay selection and interpretation.

## Conflict of Interest

The authors declare that they have no conflict of interest to disclose.

## Ethics Approval

This study was approved by the Institutional Review Board of Koç University (Approval Number: 2025.021.IRB2.017) and conducted in accordance with the ethical guidelines of the Declaration of Helsinki.

## Data Availability Statement

The datasets used and/or analyzed during the current study are available from the corresponding author on reasonable request. 
